# Changes in Pain Sensitivity in Treatment for Breast Cancer: A 12-Month Follow-Up Case Series

**DOI:** 10.3390/ijerph19074055

**Published:** 2022-03-29

**Authors:** Laura Lorenzo-Gallego, Beatriz Arranz-Martín, Helena Romay-Barrero, Virginia Prieto-Gómez, Enrique Lluch, María Torres-Lacomba

**Affiliations:** 1 Physiotherapy in Women’s Health (FPSM) Research Group, Physiotherapy Department, Faculty of Medicine and Health Sciences, University of Alcalá, 28805 Madrid, Spain; laura.lorenzo@edu.uah.es (L.L.-G.); beatriz.arranz@edu.uah.es (B.A.-M.); maria.torres@uah.es (M.T.-L.); 2 Faculty of Physiotherapy and Nursing, University of Castilla-La Mancha, 45071 Toledo, Spain; helena.romay@uclm.es; 3 Department of Physical Therapy, University of Valencia, 46010 Valencia, Spain; enrique.lluch@uv.es; 4 Pain in Motion Research Group, Department of Physiotherapy, Human Physiology and Anatomy, Faculty of Physical Education & Physiotherapy, Vrije Universiteit Brussel, 1050 Brussels, Belgium

**Keywords:** adjuvant chemotherapy, adjuvant hormonal therapy, breast neoplasm, breast surgery, pain sensitization, quantitative sensory testing, radiotherapy

## Abstract

This study aimed to investigate changes in the pain sensory profile of women with breast cancer. Five women with unilateral breast cancer were enrolled. Participants were assessed with direct (quantitative sensory testing, QST) and indirect measures of pain sensitization (self-reported central sensitization inventory, CSI) at baseline (before surgery), 1 week after surgery, and at 1, 6, 9, and 12 months post-surgery. In the event of pain occurrence, the Leeds Assessment of Neuropathic Symptoms and Signs was also used. Nociceptive pain was the predominant pain mechanism in the postoperative period, while an increase in sensitization predominated one year after breast cancer surgery, especially in those participants who had received more treatment procedures. The participants who received more therapies for breast cancer experienced persistent pain and a higher level of sensitization. An assessment protocol including direct measurements (QST) and indirect measurement (self-reported CSI) allows for detecting changes in pain sensitivity, which can be useful for characterizing and/or predicting pain before, during, and up to one year following surgical interventions for breast cancer.

## 1. Introduction

Breast cancer is the most frequent tumor and the main cause of death among women worldwide, with over 2.2 million newly diagnosed cases in 2020 [1]. However, as a result of early diagnosis and progress in the disease management, the survival rate has reached 90% over the last decade [2]. Consequently, increasing the quality of life of breast cancer survivors has become a topic of interest in the scientific literature [3]. Despite the well-demonstrated benefits of therapies for breast cancer, treatments usually imply a risk for developing adverse events in breast cancer survivors, among which pain is of relevance [3,4,5]. Along with treatments, certain individual physiological and psychosocial factors could also contribute to the development of pain during the disease [3,5,6,7,8,9].

Perceived pain in people with breast cancer changes throughout treatment. Over 50% of women suffer from severe acute pain after surgery [10] and between 11% to 60% of patients report pain occurring after radiotherapy, chemotherapy, and hormone therapy [9,11,12,13]. Pain location also shifts, starting at the breast and homolateral arm regions mainly after surgical treatment [5,8,14,15], later expanding to different body parts over time, even becoming generalized pain [16]. Additionally, an estimated 33–50% of affected women suffer from neuropathic pain following breast cancer treatments [16,17,18,19]. However, neuropathic pain is not exclusive of this population and frequently is associated with nociceptive pain, which predominates during a first stage of local–regional treatments (breast surgery and axillary lymph node dissection and/or sentinel lymph node biopsy) that are related to alterations and damage in the surrounding tissues (vascular [13], neural [17], and myofascial [20]). Pain persists in many women with breast cancer after treatment, possibly due to the sensitization of the central and peripheral nervous systems. Pain sensitization has been shown to be strongly associated with pain catastrophizing [21] and the persistent presence of pain to previous cancer treatment [22].

Quantitative sensory testing (QST) consists of a battery of psychophysical tests used to quantify the sensory perception of pain as self-reported by the patients. This allows for unraveling the mechanism(s) underlying the patient’s pain. QST is considered the gold standard for assessing changes in sensory perception because of a lesion or a disease affecting the somatosensory system [23]. It also evaluates the function of both large (A-beta) and small (A-delta and C) nerve fibers, including the corresponding central pathways. It is possible, through the application of mechanical and thermal stimuli of controlled intensity, to detect signs of sensory gains and losses [23,24]. The scope for employing QST is broad, including in the evaluation of peripheral neuropathic pain syndromes [25,26], neurological pain [27], post-surgery pain syndromes [28,29], and musculoskeletal pain [30], among others. In terms of breast cancer, different QST modalities have been employed in both pre- and post-surgery interventions [15,31,32], during and after chemotherapy treatment [33], and in the presence of persistent neuropathic pain [29] and peripheral neuropathies [34]. Among the findings of the above-mentioned studies using some of the QST psychophysical tests, the following are worth mentioning: a decrease in the pressure pain threshold (PPT) and the facilitation of temporal summation (TS), both during the post-surgical period [15] and in the presence of neuropathic pain [29], as well as a relationship of lower PPT with pain [32]; an increase in warmth detection and mechanical detection thresholds post-surgery [31], following chemotherapy [34], and in the presence of neuropathies [35]; and an increase in the vibratory detection threshold as a consequence of chemotherapy [34,35]. However, no longitudinal studies have investigated changes in pain sensitization measurements along the course of treatment in breast cancer survivors.

Thus, this study aimed to investigate changes in the pain sensory profile across breast cancer treatment in a series of women with breast cancer receiving surgery and chemotherapy, radiotherapy, and/or hormone therapy. 

## 2. Materials and Methods

### 2.1. Study Design

A longitudinal case series study was conducted between June of 2020 and June of 2021 at the Research Unit of the “Physiotherapy in Women’s Health Research Group” of the University of Alcalá (Madrid, Spain). The study protocol was approved by the Ethics Committee for Clinical Research of the Principe de Asturias Hospital. The study reporting followed the CARE guidelines (for case reports) [36]. The research followed the ethical principles of the Helsinki Declaration. All participants provided informed written consent.

### 2.2. Participants

Five women diagnosed with breast cancer recruited from the Hospital Príncipe de Asturias (Madrid, Spain) participated in the study. Women undergoing unilateral surgery with axillary lymph node dissection (ALND) or sentinel lymph node biopsy (SLNB) at the Príncipe de Asturias Hospital in Alcalá de Henares, Madrid (Spain), were considered for inclusion in the study. A decision was made to include five women in this case series, as it was a preliminary study that sought to explore: (1) changes in the pain sensory profile across the entire breast cancer treatment, and (2) the feasibility of performing the full battery of QST tests throughout breast cancer treatments.

Women without ALND or SLNB or with bilateral breast cancer, systemic disease, local/regional recurrence, neurological disorders (i.e., stroke, multiple sclerosis, peripheral nerve entrapment and injury in the upper extremity, etc.), central sensitization syndromes (i.e., fibromyalgia, irritable bowel syndrome, chronic headaches, temporomandibular disorders, pelvic pain syndromes, etc.), shoulder surgery, or previous severe pathology of the upper limbs (i.e., carpal tunnel syndrome, osteoarthritis, hand–arm vibration syndrome, etc.) were excluded. Women with cognitive impairment were also excluded when it prevented them from understanding information, answering questionnaires, and providing consent and/or participating in the trial. Finally, patients who had received previous chemotherapy or radiotherapy treatments or breast surgery were also excluded. 

### 2.3. Assessment Procedure

Each participant was assessed preoperatively and then postoperatively on hospital discharge (between day 3 and day 5), and at 1 week, 1 month, 6 months, 9 months, and 12 months after surgery. All measurements were performed by a physiotherapist with 10 years of experience in oncological therapy, who performed the tests in a peaceful environment aided by a novel physiotherapist. 

A protocolized questionnaire was devised for the recording of the women’s evaluations. Previously, a physiotherapy record file was opened, and a file number was assigned for each participant in chronological order. Data were entered in a database (Microsoft Excel), where subjects were identified by a reference number to guarantee anonymity.

During the preoperative assessment, demographic data were collected on all participants, including age, marital status, body mass index, job, educational level, and socioeconomic status. Anthropometric variables (weight and height) and menopause were recorded at all assessments. The following clinical variables were also included: former pathologies of the upper limbs, affected side, type of surgery and relevant potential complications (seroma, superficial lymphatic thrombosis, and acute pain), medical treatments (chemotherapy, radiotherapy, and hormone therapy), and breast reconstruction (yes/no and type). The onset of pain, together with its intensity and location, pharmacological treatment (yes/no, type, and dosage), sensory descriptors, and pain sensitization were also included. The examination included measures of pain sensitization. All these variables were recorded in the same manner throughout all the assessment visits.

### 2.4. Outcome Measures

#### 2.4.1. Direct Measurements of Pain Sensitization

All participants received a battery of QST following the standardized protocol described by Rolke et al. [26], which has been used on people with breast cancer before [27]. 

The following measurements were included in the QST protocol: mechanical detection threshold (MDT) and allodynia, vibration detection threshold (VDT), thermal perception of pain and onset of warm and cold stimuli, TS, PPT, and suprathreshold pressure stimulus.

##### Mechanical Detection Threshold (MDT) 

The MDT was tested using the “method of limits” through a standardized set of Von Frey filaments (Aesthesiometer, Stoelting C, Wood Dale, IL, USA) that exerts bending forces of between 0.23 and 512 mN. Series of mechanical stimuli were administered in order of descending and ascending intensity until the feeling of touch disappeared or appeared, respectively. The stimulation filaments were placed perpendicular to the medial third of the humerus in a contact area of uniform shape and size at both upper limbs, counterbalanced, and the participants were required to indicate when the monofilament touched their skin. After feeling the stimulus, subjects were asked if the perception was painful, and a positive answer was recorded as presence of allodynia.

##### Vibration Detection Threshold (VDT)

The VDT was assessed using a graduated medical tuning fork from Rydel-Seiffer (64 Hz, scale 8/8) (Valuemed^®^. Edmonton, AB, Canada) bilaterally placed three bony prominences (the epicondyle, radial styloid, and the lateral border of the acromial point) until the participant stopped feeling the vibration. The last second in which vibration was perceived was recorded as the VDT.

##### Warm and Cold Detection and Pain

For assessing warm and cold detection and pain, cold (25 °C) and hot (40 °C) stimuli were delivered bilaterally with a Rolltemp II stimulator (Somedic SenseLab AB, Sösdala, Sweden) at the posterior deltoid muscle, serratus anterior muscle, and rectus femoris muscle. Patients were asked about their perception of cold or heat as well as pain, which was quantified verbally on a numerical rating scale (NRS) if present.

##### Temporal Summation (TS) 

For the assessment of TS, a pinprick (256 mN) was applied bilaterally on the middle deltoid muscle. The perceived pain intensity with a NRS after one prick was compared against a series of 10 pricks delivered at a speed of 1 prick per second. This process was repeated five times on each arm and the wind-up ratio (WUR) was calculated by dividing the average of the five 10-prick series by the average of the five single stimuli [26]. The estimated WUR ratio was indicative of facilitated TS (WUR > 1), no changes in TS (=1), or decreased TS (WUR < 1) [37].

##### Pressure Pain Threshold (PPT)

The PPT was examined on the unaffected side via an analogue algometer (Wagner Instruments, Greenwich, CT, USA) with a round tip of 1 cm^2^. Pressure was gradually increased at a rate of 30 kPA/s and the PPT was measured at the serratus anterior muscle, the middle scalene muscle, the insertion tendon of the epicondyle muscles, and the vastus lateralis muscle. Three PPT measurements were performed at each site with a 30 s rest interval, and the mean value was used for analysis.

##### Suprathreshold Stimulus

A stimulation model of sustained pressure pain was employed for evaluating the suprathreshold pressure stimulus [38]. The same algometer as described for the PPTs was used on the infraspinatus muscle of the non-affected side at 120% of the previously calculated PPT. Following the application of this suprathreshold stimulus, participants were asked to draw on a body chart the location of pain and/or other sensations felt [38].

#### 2.4.2. Indirect Measurement of Pain Sensitization

All participants completed the self-reported Spanish-validated version of the central sensitization inventory (CSI), a screening instrument used to identify people with central sensitivity syndromes [39]. The questionnaire has two parts. Part A consists of 25 items, each ranged on a 5-point scale with the endpoints 0 = “never” and 4 = “always” (range: 0–100), which assess health-related symptoms common to central sensitization. It has a cutoff score of 40 out of 100 which is able to distinguish between individuals with central sensitivity syndromes and a non-patient comparison sample. On the other hand, part B (which is not scored) asks about the previous diagnosis of seven syndromes of central sensitization. The Spanish version of CSI has shown strong psychometric properties for subjects with chronic pain conditions [39]. 

#### 2.4.3. Pain

In the presence of pain, the following outcome measurements were also recorded to describe its occurrence: pain location, intensity via the numerical rating scale (NRS) [40], and the self-reported Leeds Neuropathic Symptoms and Signs (S-LANSS) [41].

The NRS for pain is a segmented numeric version of the visual analogue scale (VAS) that serves to measure pain intensity as well. The present study employed the 11-item NRS, where women rated their pain from 0 representing “no pain” to 10 representing “pain as bad as you can imagine”. The pain NRS provides sufficient discriminative power for chronic pain patients to describe their pain intensity [42] and is considered to be as efficient as the VAS in the assessment of pain in cancer cases [43], with reductions of 2 points or 30% in the pain scores being regarded as clinically important for overall patient improvement [43].

Finally, the self-reported S-LANSS was used to identify pain with neuropathic characteristics. It is made up of seven items, five of which inquire about pain during the last week and the other two about clinical signs of allodynia and hyperalgesia. All items present dichotomous questions (yes/no) that can be scored with values that differ among questions (0, 1, 2, 3, 5). The overall score ranges from 0 to 24, with a cutoff score of 12 out of 24 suggesting the presence of neuropathic pain. The Spanish version of the S-LANSS scale has been shown to be valid and reliable for identifying patients with chronic pain of neuropathic features [41]. 

#### 2.4.4. Data Analysis 

Descriptive data are shown for the different assessed variables for each participant. 

Changes in absolute and percentage values for each outcome between the baseline measurement (V0) and the different visits at 1 week (V1), 1 month (V2), 6 months (V3), 9 months (V4), and 12 months (V5) have been calculated and are displayed in Tables.

## 3. Results

**Figure 1:**
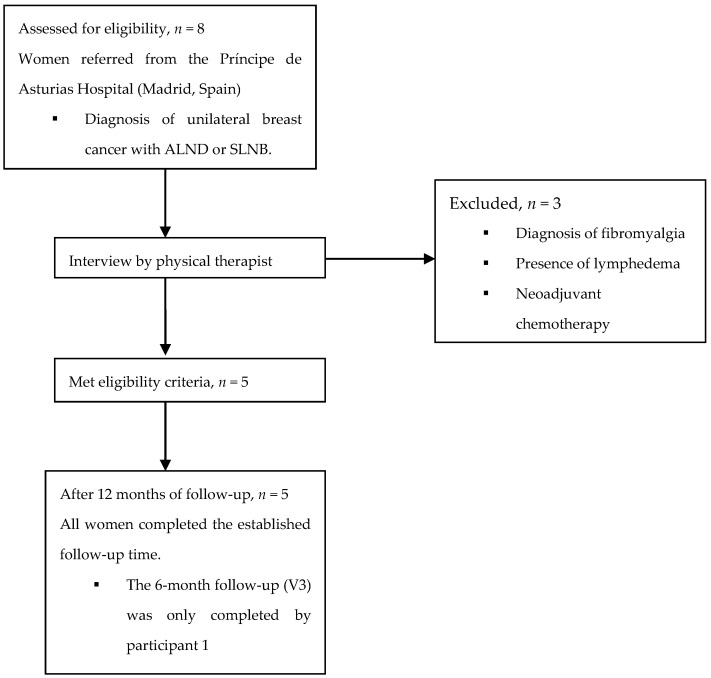
Participant flow diagram. ALND, axillary lymph node dissection; SLNB, sentinel lymph node biopsy.

Eight women diagnosed with unilateral breast cancer were assessed for eligibility. Of them, three were excluded due to the presence of lymphedema, fibromyalgia, or neoadjuvant chemotherapy, and so five participants finally completed the study (Figure 1). 

Table 1 shows the demographic and clinical characteristics as well as breast cancer treatments of the included subjects. Anthropometric measures did not change significantly throughout the study period. 

No woman attended the scheduled follow-up at V3 due to COVID−19 confinement, except for participant 1. Participant 1 did not complete any of the questionnaires at V2 for unknown reasons.

Table 2 displays changes in pain intensity and location, whenever present, throughout the different assessment points. Importantly, pain appeared immediately post-surgery (V1) in all the women and persisted at the one-year follow-up (V5) in participants 1 and 5. All participants took pain relief medication (analgesics) for one week after surgery, and participants 1 and 5 continued with their medication intake in the following assessments since the occurrence of pain persisted throughout the follow-up period.

**Figure 2:**
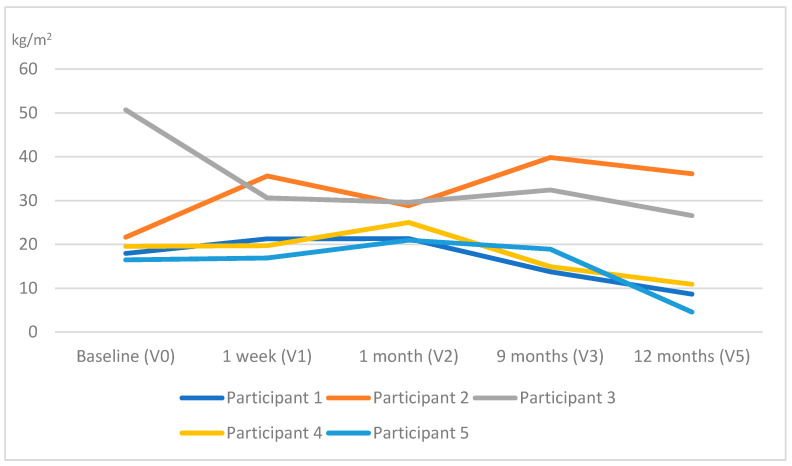
Evolution of the pressure pain threshold (PPT) throughout the study (the average PPT at the different locations has been calculated at each time point assessment for each participant).

**Figure 3:**
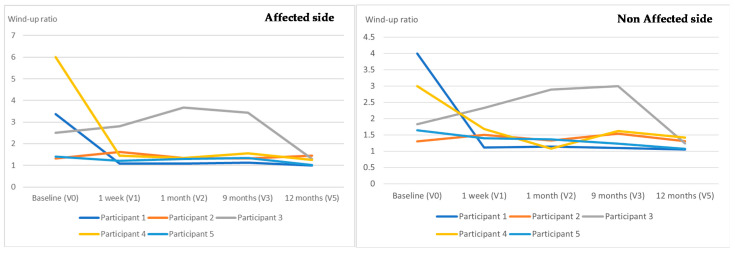
Evolution of the temporal summation throughout the study.

In terms of QST outcomes, no changes were observed across all the assessment points in the warmth detection threshold, except for participant 5. In particular, this woman presented warm anesthesia bilaterally in the posterior deltoid muscles at V4, which shifted to the rectus femoris muscle of the affected side and the serratus anterior muscle of the unaffected side at V5. Table 3 and Table 4 show the outcomes of the remaining QST outcomes for all participants. Significant reductions in all participants were observed in the PPTs (Figure 2) at all sites in all measurement points as well as a facilitated TS, which was reported bilaterally (Figure 3). Additionally, an increase in MDT and VDT was observed in participants 1 and 5, respectively, during the last assessments (V4, V5).

In terms of self-reported outcomes, highly heterogeneous results were observed among the different assessments and participants (Table 5). A significant increase in the CSI score was observed in participants 1 and 5 between the baseline (V0) and 12-month follow-up (V5) assessments. This increase even exceeded the threshold score in the aforementioned participants, who received all treatment options (surgery, chemotherapy, radiotherapy, and hormone therapy) and ended the follow-up time of suffering pain. Regarding neuropathic pain, participant 1 reached the diagnostic threshold established by the S-LANSS at V3 and participant 3 at the postsurgical visit (V1).

## 4. Discussion

The purpose of this study was to determine the changes in pain sensitivity throughout the breast cancer treatment up to one year after surgery. The obtained results showed that the more therapies are implemented (that is, having received radiotherapy plus chemotherapy, or radiotherapy plus chemotherapy plus hormone therapy in addition to surgery), the more changes in pain sensitivity are observed after breast cancer surgery. On the other hand, the dominant pain mechanism in all participants during the postoperative period was nociceptive. 

### 4.1. Direct Measurements of Sensitization

The outcomes of the VDT and MDT showed little variation. The VDT increased in participant 5 at the locations adjacent to the affected side. The MDT increased at both sides in participant 1 only following chemotherapy treatment. These findings are in agreement with those by Hershman et al. and Krøigård et al., who observed an increase in the MDT and VDT, respectively, related to the delivery of chemotherapy [33,35]. This could be due to the damage chemotherapy produces on large diameter fibers (i.e., Aβ fibers) [44,45]. 

All PPTs measured at the contralateral side of surgery gradually decreased in all participants after surgery. An increase in PPTs measured at remote sites would indicate an increase in widespread mechanical hyperalgesia, which would reveal activated pain sensitization mechanisms [30].

Changes in the suprathreshold pressure stimulus were observed at 1 week post-surgery and after 9 months. The pain feeling reported in the contralateral half of the body suggests the increased use of the unaffected arm following surgery, which could generate myofascial trigger points in the infraspinatus muscle [20,44]. 

The outcomes observed in participants 1, 2, 3, and 4 for thermal perception were inconsistent with those described in the existing literature. Krøigård et al. found abnormal values of the warmth detection threshold after receiving chemotherapy [35], similar to that observed one year after the surgery by Andersen et al. and Juhl et al. [9,31]. The current study detected an increase in the warmth detection threshold only in patient 5 at the 12-month follow-up (V5). This points to an effect on C-fibers, whose recovery is usually slower than large-caliber fibers [46]. The time point at which this effect appeared suggests a potential relationship between chemotherapy and the generated hypoesthesia [39]. 

The facilitation of TS decreased in all women at the 12-month assessment, which would indicate a lower degree of spinal cord sensitization (i.e., wind-up), despite the fact that the values of WUR remained above the facilitation threshold throughout the follow-up. Some authors have found associations between TS values and the anxiety level of patients [47], which could explain the decrease in TS values throughout the assessments, since anxiety tends to decrease by more than 15% a year after diagnosis [48]. Furthermore, although TS has been used predictively [15] and/or as a dynamic QST measure of greater pain intensity [49], no studies have been found describing the evolution of WUR over time. Participants 4 and 1, who showed the highest values of TS at the baseline, reported the greatest pain after the surgical intervention (V1). This suggests a potential positive association between an enhanced TS before surgery and the level of post-surgical pain [50], which is in agreement with the outcomes of Schreiber et al. [15] for post-mastectomy pain. The two women who developed persistent pain after surgery did not show greater facilitation of TS when compared to those without pain, contrary to what some authors have described [12,49]. The methodological differences between studies may explain this apparent discrepancy about TS. Specifically, while Schreiber et al. measured TS at the index and third finger of each hand, Gottrup et al. did not report the location where TS was calculated. In addition, Schreiber et al. performed QST on subjects who had already reported pain on the day of surgery, whereas the study by Gottrup et al. included women who presented sensory disturbances at the baseline [15,49].

### 4.2. Indirect Measurement of Sensitization

The changes in the CSI scores were clinically significant for participants 1 and 5 [39]. In particular, participant 1, who had baseline values close to sensitization, exceeded the CSI diagnostic threshold after surgery and increased her score on subsequent assessments, and participant 5 commenced to develop symptoms of central sensitization at 9 months after surgery, following chemotherapy treatment. Many studies associate medical–surgical treatments with a higher risk of suffering persistent pain [8,16,19]. In addition, other studies associate pain-related factors (i.e., pain intensity, presence of hyperalgesia, and widespread pain) throughout the treatment periods with higher CSI scores [51]. Our findings, therefore, suggest a potential relationship between the implementation of more treatment procedures and an increase in symptoms of central sensitization as measured with the CSI [16,21]. 

Furthermore, it is important to note that the cut-off score of 40/100 in the CSI was calculated in a group of patients with various central sensitization syndromes (e.g., fibromyalgia) [39], but whether this value is also applicable in breast cancer survivors is currently unknown.

### 4.3. Pain

Pain appeared in all subjects one week following the surgery (V1), mainly at the breast. This pain can be the result of tissue damage from the intervention [12,52]. After six months, the pain only persisted in participants 1 and 5. Participant 5 suffered from pain in both hands after 9 months and generalized pain at 12 months. The continuous presence of pain only in women who underwent a greater variety of treatments can be explained by the damages produced by radiotherapy and chemotherapy to the involved tissues [5,18]. The potential onset of a brachial plexopathy could be responsible for the sensory changes in the hands of participant 5 [5,53]. This woman underwent a lymphadenectomy which, together with an increase in the scarring of the intervened side, could have affected the persistence of pain in the long term [9]. Finally, the presence of generalized pain in this woman, combined with a score of 40/100 in the CSI one year post-surgery and after having undergone treatment with chemotherapy and radiotherapy (V5), likely indicates a tendency for central sensitization [54]. 

Pain intensity in participants 1 and 5 was related to adjuvant treatments different from those received by other patients [52]. Greater pain intensity was consistent with low PPT values [32]. The changes in pain sensitivity observed in these patients may be related to the implication of nerve fibers secondary to chemotherapy [35]. This could explain the occurrence of disproportionate pain or hyperalgesia, a clinical characteristic of central sensitization [16], and this increased sensitivity, in turn, could be responsible for a greater intensity of pain [32].

The pain NRS has been widely used in combination with QST, which helped to identify a relationship between pain interference and intensity [15,29,32], as was observed in the current study. Despite the high prevalence of neuropathic pain in the population with breast cancer [18,45,53,55], none of the included women ended the study with neuropathic pain as measured with the LANSS.

### 4.4. Symptomatology and Treatments

In terms of the relationships established between the observed findings and breast cancer therapies, the highest variability was observed in women who received chemotherapy and radiotherapy in addition to the other treatments (participants 1 and 5). Participant 3 underwent surgery only and did not show substantial differences compared to those receiving endocrine therapy. The literature is contradictory about the association between hormone therapy and pain, with some studies finding an association [6,16,46], while others do not [5,56,57]. However, none of the included women who were treated with hormone therapy solely developed pain during this treatment.

### 4.5. Strengths and Limitations

The main limitation of this study stems from its design, since a limited number of descriptive case series does not allow for reaching categorical conclusions and extrapolating them to the general population with breast cancer. The heterogeneity of the sample could hinder the interpretation of data, although the participants are representative of the usual clinical reality, as breast cancer treatment may include all the treatments presented in this case series, so that some women may receive only some of the treatments while others receive all the treatments in their entirety. A potential shortcoming of this study is the intake of analgesic medication when pain occurred. Finally, some follow-up assessments were incomplete for several participants, at times due to COVID−19 confinement, but in one case for unknown reasons, though likely related to lack of therapeutic adherence [58].

Among the strengths of the study is the application of a detailed protocol for the assessments that was always conducted by the same two researchers. Additionally, no studies have been found with a follow-up period as long as that of the current study, encompassing as many stages of breast cancer treatment as the current one, or conducting such an exhaustive assessment of central sensitization via both direct and indirect measurements.

### 4.6. Future Research Lines

Further longitudinal studies with adequate sample sizes are required to identify relationships between the diverse medical–surgical interventions and both direct and indirect measurements of central sensitization in order to delve into the effect of such treatments on pain sensitivity in women with breast cancer, as well as its correlation with psychosocial factors, pain, and quality of life. The evaluation of changes in pain sensitivity by subgroups of breast cancer treatments may be useful to explore not only in relation to the effect of each type of treatment on pain sensitivity, but also of each combination of treatments.

## 5. Conclusions

The participants who received more therapies for breast cancer appeared to experience persistent pain and a higher level of sensitization. An assessment protocol including direct measurements (QST) and indirect measurement (self-reported CSI) could allow for detecting changes in pain sensitivity, which would be useful for characterizing and/or predicting pain before, during, and up to one year following surgical interventions for breast cancer. A better understanding of the effects of breast cancer treatments on pain sensitivity may help to provide individualized care. 

## Figures and Tables

**Table 1:** Characteristics of the sample.

Participants					
Characteristics	1	2	3	4	5
Age (years)	51	43	36	49	39
BMI (kg/m^2^)	32.05	30.48	21.11	18.20	18.34
Menopause	Yes	No	No	No	No
Affected side	Left	Left	Right	Right	Left
Affected upper limbs	Yes	No	No	Yes	No
Previous shoulder pathology	Impingement	No	No	Painful shoulder	No
Limited motion of the shoulder	Yes	No	No	Yes	No
Number of people in the household	1	4	4	2	3
Children	0	2	2	0	0
Adults	1	2	2	2	3
Educational level	University	Non-completed secondary education	University	High school	High school
Yearly income (EUR)	<12,000	Did not answer	>48,000	12,000–24,000	24,000–36,000
Surgical procedure					
Mastectomy plus immediate reconstruction Lumpectomy	Yes No	Yes No	Yes No	No Yes	Yes No
Axillary dissection procedure					
ALND SLNB	No Yes	No Yes	No Yes	No Yes	Yes No
Postoperative therapy					
Chemotherapy	Yes	No	No	No	Yes
Radiotherapy	Yes	No	No	No	Yes
Hormone therapy	Letrozole	Letrozole	No	Letrozole	Tamoxifen

BMI, body mass index; ALND, axillary lymph node dissection; SLNB, sentinel lymph node biopsy.

**Table 2:** Pain characterization of the sample.

Participants						
Measurement	V	1	2	3	4	5
PAIN	V0	Yes	No	No	No	No
	V1	Yes	Yes	Yes	Yes	Yes
	V2	Yes	No	Yes	No	No
	V3	Yes	*	*	*	*
	V4	Yes	No	No	No	Yes
	V5	Yes	No	No	No	Yes
NRS (%)	V0	70	NP	NP	NP	NP
	V1-V0	0	10 (10)	0	80 (80)	20 (20)
	V2-V0	0	0	0	0	0
	V3-V0	0	*	*	*	*
	V4-V0	−30 (−30)	0	0	0	60 (60)
	V5-V0	−10 (−10)	0	0	0	70 (70)
LOCATION	V0	1	NP	NP	NP	NP
	V1	3	2, 5	3	3	3
	V2	3	NP	3	NP	NP
	V3	3	*	*	*	*
	V4	0	NP	NP	NP	4, 6
	V5	3	NP	NP	NP	7

V, visit; NP, no pain; NRS, numerical rating scale; Location: 0—unknown; 1—neck (trapezius area); 2—both shoulders; 3—homolateral breast; 4—both hands; 5—contralateral arm; 6—complete homolateral arm; 7—generalized body pain; * missing data.

**Table 3:** Changes (percentage) in direct measurements scores: temporal summation, suprathreshold pressure stimulus, and vibration detection threshold.

Participants							
Measurement		V	1	2	3	4	5
TS	Wind-up ratio affected side	V0	3.38	1.32	2.5	6	1.41
		V1-V0	−2.3 (−68.05)	0.31 (23.48)	0.3 (12)	−4.56 (−76)	−0.10 (−7.6)
		V2-V0	−2.31 (−68.34)	0.01 (0.76)	1.17 (46.8)	−4.65 (−77.5)	−0.11 (−7.8)
		V3-V0	−1.72 (−50.89)	*	*	*	*
		V4-V0	−2.26 (−66.86)	−0.01 (−0.76)	0.93 (37.2)	−4.45 (−74.17)	−0.08 (−5.67)
		V5-V0	−2.38 (−70.41)	0.12 (9.09)	−1.21 (−48.4)	−4.75 (−79.17)	−0.39 (−27.66)
	Wind-up ratio non-affected side	V0	4	1.3	1.83	3	1.64
		V1-V0	−2.89 (−72.25)	0.20 (15.38)	0.50 (27.32)	−1.32 (−44)	−0.23 (−15.02)
		V2-V0	−2.86 (−71.5)	0.03 (2.31)	1.06 (57.92)	−1.92 (−64)	−0.28 (−17.07)
		V3-V0	−2.4 (−60)	*	*	*	*
		V4-V0	−2.9 (−72.5)	0.24 (18.46)	1.17 (63.93)	−1.38 (−46)	−0.41 (−25)
		V5-V0	−2.95 (−73.75)	0.01 (0.77)	−0.59 (−32.24)	−1.58 (−52.67)	−0.57 (−34.76)
STPS		V0	0	0	1	3	5
		V1-V0	6 (100)	0 (0)	2 (100)	4 (100)	*
		V2-V0	0 (0)	0 (0)	0 (100)	4 (100)	0 (100)
		V3-V0	0 (0)	*	*	*	*
		V4-V0	1 (100)	0 (0)	3 (100)	4 (100)	0 (100)
		V5-V0	0 (0)	0 (0)	0 (100)	5 (100)	0 (100)
VDT	Acromion affected side (s)	V0	10	15	12	12	20.59
		V1-V0	−6 (42.86)	9 (47.37)	5 (20)	6 (35.29)	3 (16.22)
		V2-V0	3 (21.43)	0	10 (40)	−4 (−23.53)	3.39 (16.46)
		V3-V0	−1 (−7.14)	*	*	*	*
		V4-V0	−5 (−35.71)	3 (15.79)	15 (60)	7 (41.18)	1 (4.82)
		V5-V0	4 (28.57)	5 (26.32)	8 (32)	3 (17.65)	−3 (−14.57)
	Epycondile affected side (s)	V0	7	22	22	7	32
		V1-V0	−1 (−11.11)	−9 (−37.5)	−4 (−12.12)	6 (46.15)	−10.2 (−37.8)
		V2-V0	2 (22.22)	−16 (−66.67)	−7 (−21.21)	−3 (−23.08)	−12.8 (−40)
		V3-V0	0	*	*	*	*
		V4-V0	−1 (−11.11)	2 (8.32)	11 (33.33)	3 (23.08)	−20 (−62.5)
		V5-V0	−4 (−44.44)	−14 (−58.33)	−1 (−3.03)	3 (23.08)	−24 (−75)
	Styloide affected side (s)	V0	22	23	36	16	20
		V1-V0	0	2 (5.41)	−11 (−20)	12 (42.86)	0
		V2-V0	−3 (−13.64)	−1 (−2.7)	−5 (−9.09)	−9 (−32.14)	4 (16.67)
		V3-V0	−3 (−13.64)	*	*	*	*
		V4-V0	−18 (−81.82)	14 (37.84)	19 (34.55)	7 (25)	3 (12.5)
		V5-V0	−6 (−27.27)	−8 (−21.62)	−6 (−10.91)	5 (17.86)	2 (8.33)
	Acromion non-affected side (s)	V0	6	17	14	7	17
		V1-V0	−2 (−13.33)	1 (5.56)	4 (15.38)	8 (53.33)	−4.7 (−27)
		V2-V0	7 (46.67)	−7 (−38.89)	7 (26.92)	−4 (−26.67)	−6.8 (−40)
		V3-V0	1 (6.67)	*	*	*	*
		V4-V0	−1 (−6.67)	−3 (−16.67)	12 (46.15)	6 (40)	−6 (−35.29)
		V5-V0	9 (60)	−10	4	5	−8 (−47.06)
	Epicondyle non-affected side (s)	V0	6	17	14	7	17
		V1-V0	4 (38.36)	2 (14.29)	−4 (−13.79)	2 (15.5)	−10.4 (−61.17)
		V2-V0	2 (18.18)	−4 (−28.57)	−4 (−13.79)	−8 (−50)	−14.1 (−55.53)
		V3-V0	0	*	*	*	*
		V4-V0	−1 (−9.09)	2 (14.29)	10 (34.48)	6 (37.5)	−15.39(−6.61)
		V5-V0	−3 (−27.27)	−6 (−42.86)	5 (17.24)	5 (31.25)	−16.39 (−64.65)
	Styloide non-affected side (s)	V0	14	23	32	15	12.4
		V1-V0	9 (88.46)	1 (2.94)	−4 (−10.53)	10 (7.03)	−0.44 (−3.54)
		V2-V0	12 (100)	1 (2.94)	0	−6 (−22.22)	−0.91 (−4.79)
		V3-V0	5 (73.08)	*	*	*	*
		V4-V0	−8 (23.08)	11 (32.35)	6 (15.79)	12 (44.44)	5.6 (29.47)
		V5-V0	5 (73.08)	−11 (−32.35)	−2 (−5.26)	10 (37.03)	6.6 (3.74)

V, visit; TS, temporal summation; STPS, suprathreshold pressure stimulus; VDT, vibration detection threshold. STPS, suprathreshold pressure stimulus location: 0—local pain; 1—pain irradiated to the homolateral shoulder; 2—numbness in the homolateral arm; 3—pain in the homolateral scapular area; 4—pain in the homolateral arm; 5—cramp in the complete homolateral arm; 6—pain in the homolateral neck area; * missing data.

**Table 4:** Changes (percentage) in direct measurements scores: mechanical detection threshold and pressure pain threshold.

Participants							
Measurement		V	1	2	3	4	5
MDT	Affected side increasing (g)	V0	0.16	0.6	1	2	0.4
		V1-V0	0	0.4 (40)	0	0	0
		V2-V0	0.24 (60)	−0.44 (−44)	−0.84 (−84)	0	−0.36 (−90)
		V3-V0	0	*	*	*	*
		V4-V0	−0.12 (−30)	0	−0.6 (−60)	−1.4 (−70)	0
		V5-V0	0.24 (60)	0.4 (40)	−0.6 (−60)	−1.4 (−70)	0
	Affected side decreasing (g)	V0	0.16	0.16	0.6	0.6	0.16
		V1-V0	15.84 (99)	0.24 (40)	−0.44 (−73.33)	1.4 (70)	0.24 (40)
		V2-V0	−0.09 (−0.56)	0.24 (40)	−0.44 (−73.33)	0.4 (20)	−0.152 (−95)
		V3-V0	−0.09 (−0.56)	*	*	*	*
		V4-V0	−0.14 (−0.88)	0.44 (73.33)	−0.44 (−73.33)	−0.2 (−10)	0
		V5-V0	−0.09 (−0.56)	0.24 (40)	−0.44 (−73.33)	−0.2 (−10)	0
	Non-affected side increasing (g)	V0	0.16	0.4	1	1.4	0.16
		V1-V0	0.24 (60)	0	−0.6 (−60)	−1.24 (−88.57)	0.12 (30)
		V2-V0	−0.12 (−30)	0	−0.84 (−54)	−1 (−71.43)	0.24 (60)
		V3-V0	0.24 (60)	*	*	*	*
		V4-V0	−0.12 (−30)	0.2 (33.33)	−0.6 (−60)	−0.8 (−57.14)	0
		V5-V0	0.24 (60)	0.2 (33.33)	−0.6 (−60)	−1 (−71.43)	0
	Non-affected side decreasing (g)	V0	0.07	0.16	0.6	0.6	0.16
		V1-V0	0.09 (22.5)	−0.09 (−22.5)	−0.53 (−88.33)	0.1 (14.29)	−0.09 (−56.25)
		V2-V0	−0.03 (−7.5)	0	−0.53 (−88.33)	−0.2 (−28.57)	−0.09 (−56.25)
		V3-V0	0.33 (82.5)	*	*	*	*
		V4-V0	−0.05 (−12.5)	0.24 (60)	−0.44 (−73.33)	−0.44 (−62.86)	0
		V5-V0	0	0.24 (60)	−0.44 (−73.33)	−0.44 (−62.86)	0
PPT	Anterior serratus (kg/m^2^)	V0	16	30.17	52	18.67	10.33
		V1-V0	4.1 (17.21)	2.56 (6.29)	−21.33 (−41.02)	−5.34 (−27.63)	−0.21 (−0.10)
		V2-V0	7.83 (32.86)	−3.67 (−9.02)	−28 (−53.85)	0.66 (3.41)	9.67 (48.35)
		V3-V0	−1.5 (−6.29)	*	*	*	*
		V4-V0	−3 (−12.59)	10.5 (25.82)	−9.33 (−17.94)	−11.34 (−58.67)	2 (10)
		V5-V0	−10.33 (−43.35)	−3.17 (−7.79)	−27 (−51.92)	−11.67 (−60.37)	−4.66 (−23.3)
	Epicondyles (kg/m^2^)	V0	20.5	37.33	49.67	18.33	20
		V1-V0	1.17 (4.94)	−7.66 (−18.68)	−15.34 (−30.88)	4.34 (17.36)	−0.42 (−0.19)
		V2-V0	3.17 (13.29)	−9.33 (−22.76)	−21.84 (−43.97)	6.67 (26.68)	−2.17 (−10.01)
		V3-V0	−0.5 (−2.11)	*	*	*	*
		V4-V0	−7.5 (−31.9)	3.67 (8.95)	−25 (−50.33)	0.67 (2.68)	1.67 (7.71)
		V5-V0	−15.17 (−64.09)	−4.5 (−10.98)	−23.34 (−46.99)	−1.66 (−6.64)	−13 (−59.99)
	Vastus lateralis quadriceps (kg/m^2^)	V0	25.33	0	53.33	29.33	23.33
		V1-V0	4 (13.04)	64.67 (91.95)	−11.66 (−21.86)	0.5 (1.27)	−23.33 (−71.41)
		V2-V0	5.34 (17.41)	44.83 (63.74)	−7.5 (−14.06)	10 (25.43)	9.34 (28.59)
		V3-V0	−5.33 (−17.38)	*	*	*	*
		V4-V0	−6.33 (−20.64)	64.33 (91.47)	−19.33 (−36.25)	−6 (−15.26)	7.67 (23.48)
		V5-V0	−5.66 (−18.45)	70.33 (100)	−19.33 (−36.25)	−11.33 (−28.81)	−21 (−64.28)
	Medium scalene (kg/m^2^)	V0	10	19.07	47.67	11.83	12.17
		V1-V0	4 (28.57)	−3.74 (−19.61)	−32 (−67.13)	1.17 (7.16)	−0.67 (−11.5)
		V2-V0	−3 (−21.43)	−3.07 (−16.1)	−27 (−56.64)	4.5 (27.56)	1.16 (8.7)
		V3-V0	−3.33 (−23.79)	*	*	*	*
		V4-V0	0	−5.74 (−30.1)	−19.34 (−40.57)	−1.83 (−11.21)	−1.5 (−11.25)
		V5-V0	−6 (−42.86)	−4.74 (−24.86)	−26.67 (−55.95)	−9.83 (−60.2)	−8.84 (−66.32)

V, visit; MDT, mechanical detection threshold; PPT, pressure pain threshold; * missing data.

**Table 5:** Changes (percentage) in self-reported measurements scores.

Participants						
Questionnaire	V	1	2	3	4	5
CSI (0–100)	V0	40	23	29	28	25
	V1-V0	9 (9)	−14 (−14)	−13 (−13)	7 (7)	4 (16)
	V2-V0	*	−14 (−14)	−18 (−18)	−2 (−2)	6 (6)
	V3-V0	2 (2)	*	*	*	*
	V4-V0	17 (17)	−5 (−5)	−19 (−19)	*	20 (20)
	V5-V0	9 (9)	−14 (−14)	−14 (−14)	5 (5)	15 (15)
LANSS (0–24)	V0	2	NP	NP	0	NP
	V1-V0	9	2	15	10	2
	V2-V0	*	0	8	0	0
	V3-V0	15	*	*	*	*
	V4-V0	1	0	0	*	10
	V5-V0	1	0	0	0	10

V, visit; NP, no pain; CSI, central sensitization inventory; LANSS, Leeds Assessment of Neuropathic Symptoms and Signs; * missing data.

## Data Availability

Data are held securely by the research team and may be available upon reasonable request and with relevant approvals in place.
